# The Significance of Circulating Microbial Signatures in the Prognosis and Immune Microenvironment of Patients with Cervical Cancer

**DOI:** 10.3390/ijms26094293

**Published:** 2025-05-01

**Authors:** Huakai Wen, Yumeng Zhang, Yongwei Liu, Haixia Long, Yuhua Yao

**Affiliations:** 1 School of Mathematics and Statistics, Hainan Normal University, Haikou 570100, China; 202212070100017@hainnu.edu.cn (H.W.); zhangym@geneis.cn (Y.Z.); liuyongwei2025@163.com (Y.L.); 2 College of Information Science Technology, Hainan Normal University, Haikou 571158, China; myresearch_hainnu@163.com; 3 Key Laboratory of Data Science and Intelligence Education, Ministry of Education, Hainan Normal University, Haikou 570100, China; 4 Key Laboratory of Computational Science and Application of Hainan Province, Hainan Normal University, Haikou 570100, China

**Keywords:** circulating microbiome, tumor immune microenvironment, prognostic biomarker, drug sensitivity

## Abstract

An increasing body of research indicates that the circulating microbiome plays a significant role in cancer initiation and progression and the treatment response. The genomic characteristics of circulating microorganisms may influence the tumor immune microenvironment, thereby affecting cancer progression and therapeutic outcomes. However, whether the circulating microbiome can serve as a prognostic biomarker for cervical cancer patients and its mechanistic role in the tumor immune microenvironment still requires further investigation. Univariate, Lasso, and multivariate Cox regression analyses were utilized to identify the circulating microbial signatures associated with overall survival (OS) in patients with cervical cancer. A circulating Microbial Abundance Prognostic Score (MAPS) model was constructed based on these findings. A nomogram that integrated clinical features and MAPSs was developed to predict the OS rates in patients with cervical cancer. Blood microbiome data were combined with matched tumor RNA-seq data to analyze the differences in the tumor microenvironment between high- and low-MAPS groups, elucidating the impact of the MAPS on the tumor immune microenvironment. Finally, the potential application of the circulating MAPS to predicting the efficacy of immunotherapy and chemotherapy was assessed. The MAPS predictive model, which includes 15 circulating microorganisms, has shown independent prognostic value for patients with cervical cancer. Integrating the MAPS into a nomogram improved the accuracy of the prognostic predictions. Combined microbial and gene analyses revealed potential interactions between prognostic tumor microbiomes and the tumor immune microenvironment. The drug sensitivity analysis indicated the potential of MAPS as a predictor of chemotherapy’s efficacy. Our findings suggest that circulating microbial signatures hold promise as novel prognostic biomarkers and may inform personalized treatment strategies in cervical cancer. Further large-scale and multicenter studies are warranted to validate the clinical utility of the MAPS.

## 1. Introduction

Cervical cancer (CESC) constitutes a pressing global health burden, ranking as the third most prevalent and lethal gynecological malignancy worldwide in both its incidence and mortality rates [1]. The traditional research has mainly focused on the impact of the vaginal microbiome (VMB) on the pathological processes in the pathogenesis of cervical cancer (CESC), with persistent infection by high-risk human papillomavirus (HPV) being recognized as a key etiological factor for this disease [2,3,4,5]. In recent years, with the advancement of microbiome research, there has been growing recognition of the potential role of the circulating microbiome in cancer initiation and progression and immune regulation [6,7,8].

The circulating microbiome refers to the bacteria, viruses, and fungi and their metabolites present in the bloodstream. These microorganisms can enter the blood circulation through various pathways, such as disruption of the gut barrier, oral infections, skin wounds, and release from tumor tissues [6,9,10]. Studies have shown that the circulating microbiome can not only influence the host metabolism as distal signaling molecules but may also affect cancer development by modulating systemic immune responses [11]. Existing research has demonstrated that the circulating microbiome exhibits characteristic compositional patterns in several cancers, including early-onset breast cancer, prostate cancer, colorectal cancer, pancreatic cancer, and hepatocellular carcinoma [12,13,14,15]. The vast microbiota in the host not only help maintain host immune balance but also play a crucial role in shaping the tumor microenvironment, thereby influencing tumor progression and the response to therapy [8,16,17,18].

Although the relationship between the circulating microbiome and various cancers is gradually being recognized, its specific role in cervical cancer remains unclear, and relevant studies are still limited. Given the recognized impact of the microbiome on the tumor immune microenvironment, exploring the potential role of the circulating microbiome in cervical cancer is significant. This study aims to construct a circulating Microbial Abundance Prognostic Score (MAPS) model based on the abundance features of the circulating microbiome. The scoring system of this model will be used to group patients, and combined with tumor immune microenvironment characteristics, its potential application to treatment response and patient prognoses will be assessed. This study is expected to provide new insights into the interaction between the circulating microbiome and the immune system, offering theoretical support for optimizing cancer treatment strategies.

## 2. Results

### 2.1. Circulating Microbiome Features Associated with Cervical Cancer Prognosis

**Figure 1:**
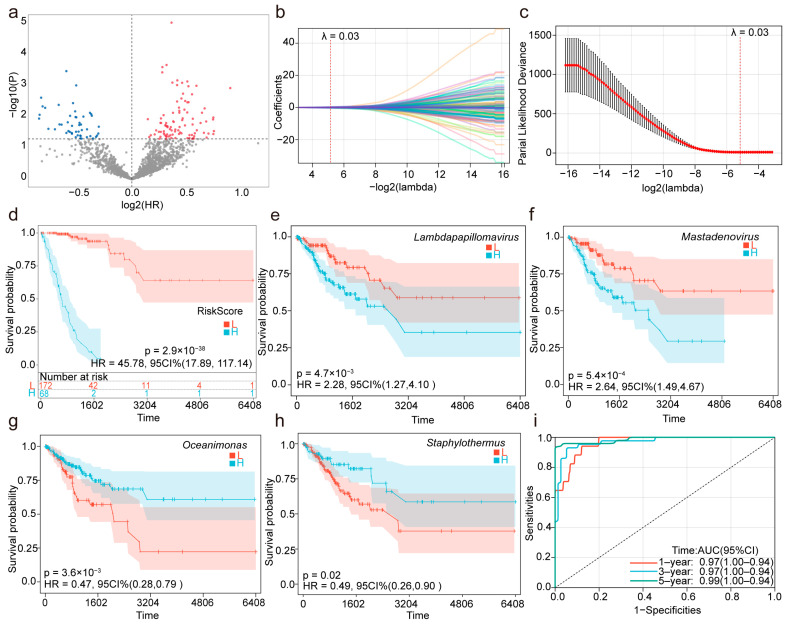
Construction and evaluation of a microbiome-associated prognostic model for cervical cancer patients. (**a**) A volcano plot illustrating candidate microbial features significantly associated with overall survival (OS) based on the univariate Cox regression analysis. (**b**) The selection of 42 microbial features using Least Absolute Shrinkage and Selection Operator (Lasso) regression. (**c**) Determination of the optimal lambda value for Lasso regression. (**d**) Kaplan–Meier survival curve for MAPS. (**e**–**h**) Kaplan–Meier survival curves for representative microbial features (*Lambdapapillomavirus*, *Mastadenovirus*, *Oceanimonas*, and *Staphylothermus*). (**i**) Receiver operating characteristic (ROC) curves evaluating the predictive performance of the MAPS for 1-year, 3-year, and 5-year survival rates.

To investigate the prognostic value of circulating microbiome features in patients with cervical cancer, TCGA’s cervical cancer circulating microbiome dataset was analyzed. A total of 1553 genera were detected across 240 samples. Initially, the univariate Cox regression analysis identified 142 genera significantly associated with the survival of cervical cancer (CESC) patients (Figure 1a). Among these, 94 microbial genera were identified as risk factors (hazard ratio [HR] > 1, *p* < 0.05), while 48 genera were considered protective factors (HR < 1, *p* < 0.05). To refine the prognostic signature further and prevent overfitting, a Lasso regression analysis was applied using the “glmnet” (version 4.1.8) package in R. The optimal lambda (penalty parameter) was determined via 10-fold cross-validation by selecting the value that minimized the partial likelihood deviance (λ. min). In the context of Lasso regression, the penalty parameter (lambda) plays a crucial role in controlling the complexity of the model. The lambda parameter penalizes large regression coefficients, thereby reducing the influence of less relevant genera and promoting sparsity. By applying cross-validation, we identified the lambda value that struck the best balance between the model’s fit and generalizability, ensuring that only the most relevant genera were retained in the model. The lambda that minimized the partial likelihood deviance was selected, effectively narrowing down the list of survival-related genera. This approach helped us to identify the 42 genera with the strongest prognostic relevance while preventing overfitting. Genera with non-zero coefficients under this lambda were retained, resulting in 42 survival-related genera (Figure 1b,c). Subsequently, a multivariate Cox regression analysis was conducted using these 42 genera. After adjusting for confounding factors, 15 genera remained statistically significant (*p* < 0.05) and were considered independent prognostic indicators for patient survival (Table 1). Among these, eight microbial genera—*Halonatronum*, *Mastadenovirus*, *Lambdapapillomavirus*, *Stanieria*, *Catonella*, *Amycolatopsis*, *Leifsonia* and *Rhodothermus*—were identified as prognostic risk factors, while seven genera—*Aureimonas*, *Apibacter*, *Staphylothermus*, *Xylella*, *Oceanimonas*, *Anaplasma* and *Saccharomonospora*—were identified as prognostic protective factors.

Furthermore, this study constructed a circulating Microbial Abundance Prognostic Score (MAPS) model by linearly combining the abundance of the 15 microbial genera with their multivariate Cox regression coefficients. This model was utilized to assess the mortality risk of patients.

The optimal cutoff value for the MAPS was calculated using the “maxstat” (version 0.7.25) package in R, with the minimum group size set to greater than 25% and the maximum group size set to less than 75%. The final optimal cutoff value was determined to be −24.843859, based on which patients were stratified into the high- and low-MAPS groups. Prognostic differences between the two groups were analyzed further using the “survfit” function from the “survival” (version 3.7.0) package in R, and the significance of the prognostic differences between groups was assessed using the log-rank test. The Kaplan–Meier curves and log-rank test results demonstrated that the survival rate of the CESC patients in the high-MAPS group was significantly lower than that in the low-MAPS group (Figure 1d). Among the 15 microbial genera identified through the multivariate Cox regression analysis, we illustrated the impact of four genera—Lambdapapillomavirus, Mastadenovirus, Oceanimonas, and Staphylothermus—on survival (Figure 1e–h). These genera were selected based on their strong statistical significance and high prognostic weight in the model, as reflected by their Cox regression coefficients (Table 1). Among them, *Mastadenovirus* demonstrated potential in subsequent discussions regarding its prognostic and biological significance in cervical cancer. This focused illustration highlights representative prognostic features while ensuring the clarity and interpretability of the results. Finally, the predictive performance of the MAPS for the 1-year, 3-year, and 5-year survival rates was evaluated using a ROC analysis with the “pROC” (version 1.18.5) package in R. The area under the curve (AUC) values were 0.97, 0.97, and 0.99, respectively (Figure 1i). To validate the MAPS model further, we constructed both a Random Survival Forest (RSF) and a DeepSurv model using the same 15 microbial features. The AUCs of the RSF model for the 1-year, 3-year, and 5-year survival predictions were 0.82, 0.78, and 0.79, respectively. The DeepSurv model achieved AUCs of 0.97, 0.95, and 0.97 at the corresponding time points (Figure S1). Compared to these models, the MAPS model demonstrated a superior or comparable prognostic performance, indicating its high robustness and predictive power.

For the details of the RSF and DeepSurv models, see Supplementary Materials S2 and S3.

### 2.2. MAPS as an Independent Prognostic Indicator for Patients with Cervical Cancer

**Figure 2:**
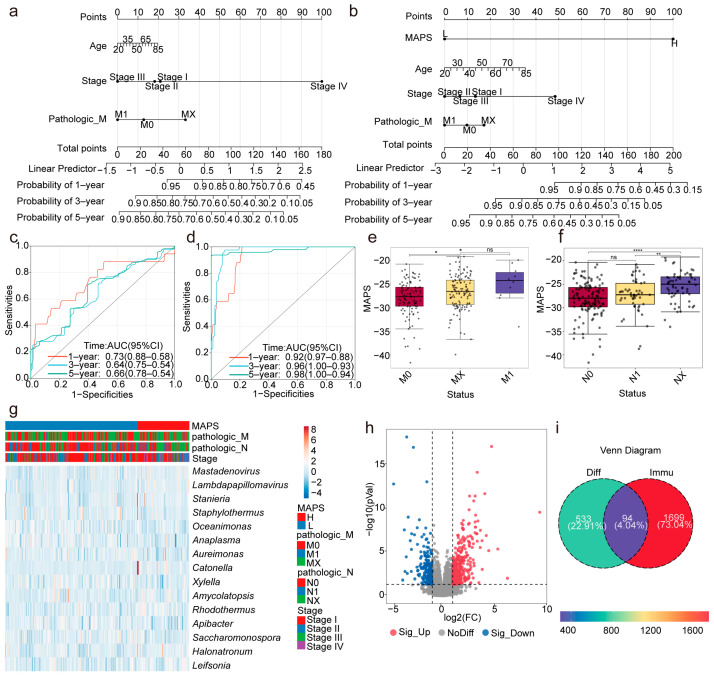
Construction of the MAPS-based prognostic nomogram for cervical cancer patients and its association with clinical characteristics. (**a**) The nomogram model based on clinical factors without the MAPS for predicting the 1-, 3-, and 5-year survival probabilities in cervical cancer patients. (**b**) Comparison with A: a nomogram model with the MAPS and clinical factors for predicting the 1-, 3-, and 5-year survival probabilities in cervical cancer patients. (**c**) The time-dependent ROC analysis showing the performance of the nomogram models without the MAPS. (**d**) The time-dependent ROC analysis showing the performance of nomogram models with the MAPS. (**e**,**f**) Box plots showing the relationship between the MAPS and clinical characteristics in cervical cancer patients (**** *p* < 0.0001, ** *p* < 0.01, * *p* < 0.05, ns = not significant, *p* > 0.05). (**g**) A heatmap illustrating the relationships between 15 microbial features and various clinical characteristics. (**h**) A volcano plot showing differentially expressed genes (DEGs) between high- and low-MAPS groups in tumors, as determined by DESeq2 (*p* < 0.05, |log2FC| > 1). (**i**) A Venn diagram depicting the overlap between differential genes and immune genes in the ImmPort database.

Utilizing the MAPSs and the clinical features (including age, tumor stage, and M stage), a multivariate Cox regression analysis was conducted to assess whether the MAPS could serve as an independent prognostic indicator. By integrating the clinical factors without the MAPS (Figure 2a) and with the MAPS (Figure 2b), a nomogram survival model was developed for predicting the 1-year, 3-year, and 5-year survival probabilities of patients with cervical cancer. In this study, we employed the “rms” (version 6.8.1) package in R to construct a nomogram model by integrating the survival time, survival status, MAPS, and clinical factors (tumor stage, M stage, and age) using the Cox regression method. The prognostic significance of these features was evaluated across 240 samples. The concordance index (C-index) for the model incorporating the MAPS was 0.90, whereas the C-index for the model without the MAPS was 0.66 (Figure 2c,d). Time-dependent ROC curves demonstrated that the MAPS significantly improved the prognostic predictive performance. In summary, the circulating microbiome is closely associated with the prognosis of patients with cervical cancer, and the MAPS model, comprising 15 microbial genera, holds significant value in prognostic prediction.

### 2.3. The MAPS Is Closely Associated with the Clinical Features of Patients with Cervical Cancer

To explore the relationship between the MAPS and the clinical features of patients with cervical cancer further, this study stratified patients into high- and low-MAPS groups based on their MAPSs. Differences in the clinical features between the two groups were analyzed. The results revealed that the MAPSs were significantly correlated with several key clinical features, including the patients’ pathological M stage, pathological N stage, and pathological T stage (Figure 2e,f and Figure S2). Additionally, a heatmap was constructed to visualize the abundance patterns of the 15 prognostic microbiota genera across all samples, with the samples ordered by MAPS and annotated with clinical variables including M stage, N stage, and overall clinical stage, highlighting the potential associations between the microbial features and clinical characteristics. These analyses indicate that MAPSs are closely associated with cervical cancer prognosis. The patients in the high-MAPS group tended to exhibit poorer prognoses, suggesting that the MAPS may serve as a potential prognostic indicator. Furthermore, the potential of this scoring system for clinical application warrants further exploration in future studies to guide personalized treatment strategies for patients with cervical cancer. Collectively, these results demonstrate that the MAPS is closely linked to the clinical features of patients with cervical cancer and may play a significant role in the clinical management of the disease.

### 2.4. The Integrated Analysis Reveals Potential Immune–Microbial Interactions

Based on the matched tumor RNA-seq data from patients with cervical cancer, 627 differentially expressed genes (DEGs) (*p* < 0.05, |log2FC| > 1) were identified between the high-MAPS and low-MAPS groups, effectively distinguishing the two groups (Figure 2h).

Additionally, based on the ImmPort database, 94 immune-related differentially expressed genes (*p* < 0.05, |log2FC| > 1) were identified in the tumors between the high- and low-MAPS groups (Figure 2i).

**Figure 3:**
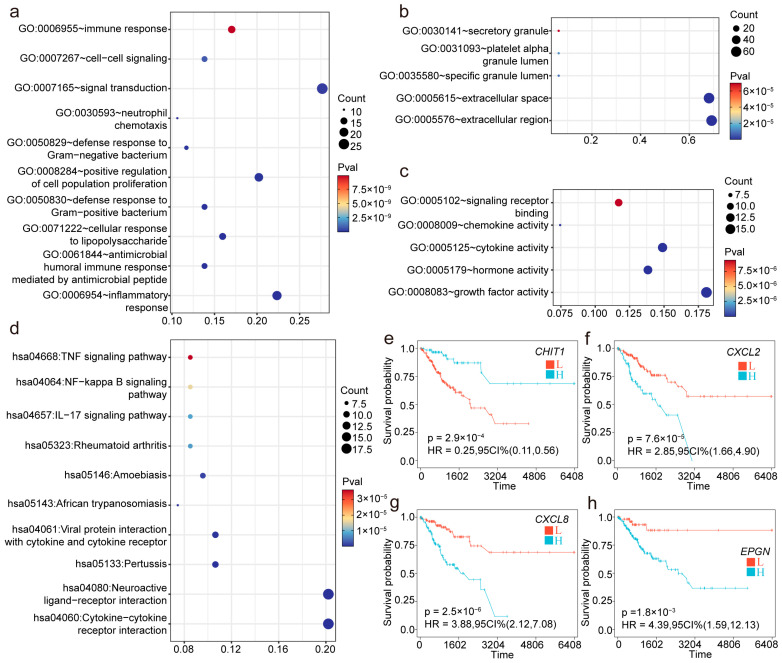
Functional enrichment analysis and survival predictions of immune-related differentially expressed genes (DEGs). (**a**–**d**) GO and KEGG enrichment analyses based on immune-related DEGs. (**e**–**h**) Kaplan–Meier survival curves for representative immune-related DEGs (*CHIT1*, *CXCL2*, *CXCL8*, and *EPGN*).

The GO and KEGG enrichment analyses revealed that these immune-related differentially expressed genes (DEGs) are extensively involved in multiple biological processes and signaling pathways. Specifically, as shown in Figure 3a, the DEGs are significantly associated with immune responses, cell signaling, chemokine activity, and cytokine–cytokine receptor interactions, indicating their important roles in immune regulation and intercellular communication. Moreover, the GO analysis in Figure 3b highlights enrichment in processes related to secretory granules and extracellular regions, suggesting that these genes may participate in secretion or extracellular signaling modulation. Figure 3c demonstrates that these DEGs are also enriched in receptor binding and cytokine activity, further emphasizing their critical roles in signal transduction and immune modulation. The KEGG pathway analysis shown in Figure 3d reveals the significant enrichment of DEGs in inflammation- and immunity-related pathways, including the TNF signaling pathway, the NF-kappa B signaling pathway, and the IL-17 signaling pathway. These findings support the potential involvement of DEGs in immune diseases and inflammatory responses. Collectively, these results suggest that these DEGs play key roles in immune responses, signal transduction, cytokine activity, and inflammatory processes and may provide new insights into disease mechanisms and potential therapeutic targets (Figure 3a–d). Furthermore, the univariate Cox regression analysis combined with the Kaplan–Meier survival analysis of the immune-related DEGs identified 11 immune-related DEGs significantly associated with the survival of patients with cervical cancer (Table 2).

Among these, CHIT1, FAM3B, and IL12B were identified as favorable prognostic factors, while CXCL2, IL1A, EREG, TL1B, STC1, CXCL8, EPGN, and TNF were identified as prognostic risk factors (Figure 3e–h and Figure S3).

**Figure 4:**
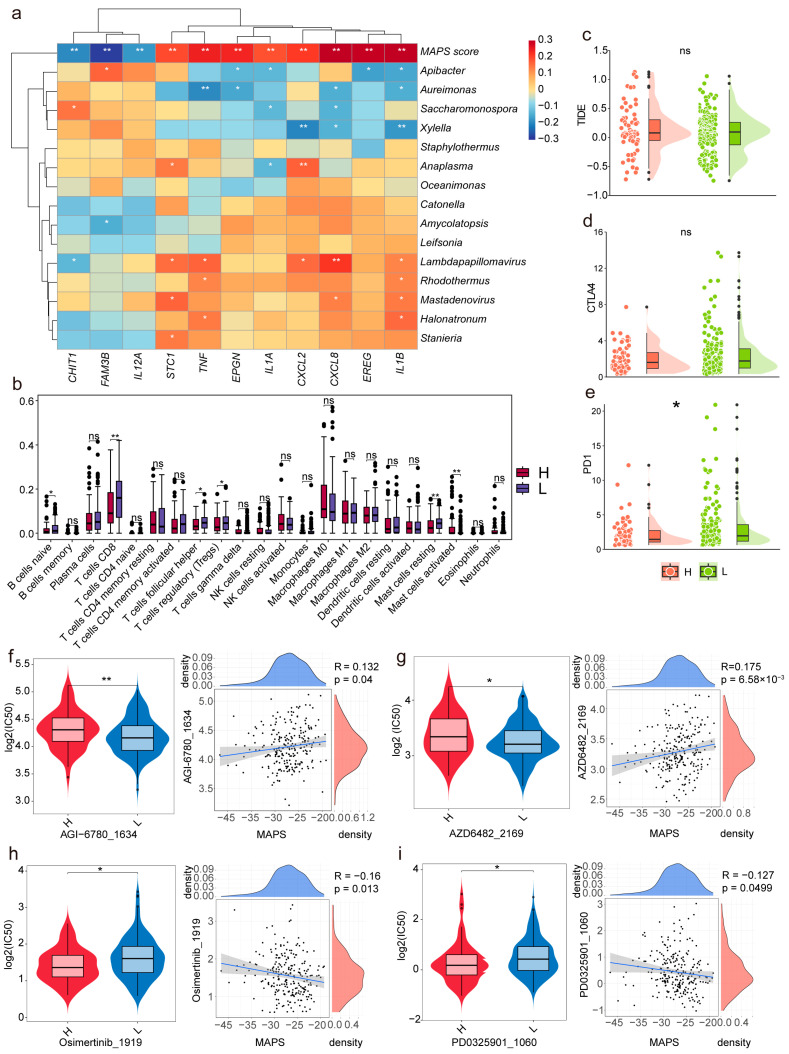
Association analysis of MAPS with immune microenvironment, immune checkpoints, and drug sensitivity (** *p* < 0.01, * *p* < 0.05, ns = not significant, *p* > 0.05). (**a**) Heatmap illustrating the relationships among the abundance of 15 microbial species, MAPS, and 11 survival-associated immune-related DEGs. (**b**) Immune infiltration analysis performed using CIBERSORT and visualized using box plots. (**c**–**e**) Differential expression and variation of TIDE, PD-1, and CTLA4 between the high- and low-MAPS groups. (**f**–**i**) Box plots and correlation plots comparing the IC50 values of AGI-6780_1634, AZD6482_2169, Osimertinib_1919, and PD0325901_1060 between the high- and low-MAPS groups.

The correlation heatmap (Figure 4a) between the 15 microbial genera, the MAPSs, and 11 survival-related immune DEGs revealed that the MAPSs exhibited strong positive correlations with IL1B, CXCL8, and EREG while showing a strong negative correlation with FAM3B. Additionally, the immune infiltration analysis conducted using CIBERSORT (Figure 4b) demonstrated a significant difference in the CD8+ T-cell infiltration between the high- and low-MAPS groups, with a higher expression observed in the low-MAPS group. Higher levels of infiltration of CD8+ T cells are generally associated with better prognostic outcomes, which is reflected in the low-MAPS group. The differential characteristics of the MAPS in the immune infiltration analysis provide significant theoretical foundations and potential research directions for subsequent studies on immune checkpoint inhibitors and drug sensitivity analyses.

### 2.5. The Impact of MAPS Expression on Immune Checkpoints and Drug Sensitivity

Recent studies have demonstrated that the microbiota play a significant role in modulating responses to cancer therapies. Patients with high expression of PD-1 or CTLA-4 may derive greater benefits from immunotherapy [19]. In this study, we found a significant difference in the expression levels of the PD-1 immune checkpoint molecule between the high-MAPS and low-MAPS groups. Therefore, the predictive value of the circulating MAPS for immunotherapy was explored through a TIDE (Tumor Immune Dysfunction and Exclusion) immune therapy response assessment. However, no significant differences were observed between the high-MAPS and low-MAPS groups (Figure 4c–e). This suggests that the MAPS may have limited utility in predicting the efficacy of immunotherapy.

To elucidate the relationship between MAPS and drug sensitivity further, we predicted the IC50 values of various drugs in patients with cervical cancer (CESC) using the “oncoPredict” (version 1.2) package. The results indicated significant differences in the therapeutic efficacy of certain drugs between the high- and low-MAPS groups. This study found that the IC50 values for six drugs—AGI-6780_1634, AZD6482_2169, Daporinad_1248, Tozasertib_1096, MK-2206_1053, and Navitoclax_1011—were significantly higher in the high-MAPS group compared to those in the low-MAPS group (Figure 4f,g and Figure S2), indicating that the patients with CESC in the high-MAPS group exhibited greater resistance to these drugs. Conversely, the IC50 values for seven drugs—Osimertinib_1919, PD0325901_1060, SCH772984_1564, Selumetinib_1736, Dasatinib_1079, Ulixertinib_1908, and WIKI4_1940—were significantly higher in the low-MAPS group compared to those in the high-MAPS group (Figure 4h,i and Figure S4), suggesting that patients with CESC with high MAPSs may benefit more from these drugs.

## 3. Discussion

In recent years, research on novel biomarkers has primarily focused on the in-depth analysis and exploration of genomic and proteomic profiles [20,21]. However, with the rapid development of high-throughput sequencing technologies and continuous improvements in data analysis tools, the role of the circulating microbiota in cancer has been more thoroughly investigated. These technological advancements have provided robust support for further progress in this field [22,23]. Studies have shown that the potential role of microbiota in cancer is becoming increasingly prominent, with their critical involvement in cancer diagnosis and pathogenesis and the treatment of malignant tumors receiving widespread attention [24,25,26,27,28]. Additionally, recent research on breast cancer patients has revealed that the features of the microbiome are closely associated with breast cancer prognosis and exhibit significant clinical predictive value [29]. Relevant circulating microbiota have been confirmed as reliable prognostic tools for patients with nasopharyngeal carcinoma and provide a basis for the treatment decisions in patients with varying degrees of malignant progression [30].

Through a systematic analysis of the features of the microbiome, this study revealed a potential association between the circulating microbiota and cervical cancer progression, successfully constructing a circulating Microbial Abundance Prognostic Score (MAPS) model based on 15 circulating microbial genera. This model demonstrated significant correlations with patients’ overall survival (OS) and exhibited a robust prognostic predictive performance when validated in an independent cohort, providing a novel tool for individualized prognosis assessments in patients with cervical cancer. Further research indicated that the characteristic microorganisms in the MAPS may influence the disease progression by modulating the tumor immune microenvironment [31]. Notably, this study proposed that the MAPS could serve as a potential biomarker for predicting the sensitivity to chemotherapy in patients with cervical cancer, offering new insights for optimizing treatment strategies. These findings contribute to a deeper understanding of the mechanisms underlying immune–microbial interactions during tumor initiation and progression while providing theoretical foundations and potential intervention targets for precision medicine in cervical cancer. For example, we observed that the expression of immune checkpoint genes, such as PD-1, differed significantly between the high- and low-risk MAPS groups, suggesting potential differences in immune regulation. However, the TIDE analysis did not reveal a significant difference in the predicted response to immunotherapy between the two groups, indicating that risk stratification alone may have limitations in forecasting immunotherapeutic efficacy. Nevertheless, the IC50-based drug sensitivity analysis revealed significant differences in the responses to several targeted agents between the MAPS groups, suggesting the potential role of MAPS in guiding personalized targeted therapies. These findings suggest that while the MAPS may offer insights into the immune landscape and therapeutic response, further validation is warranted to clarify its role in clinical decision-making.

To interpret the biological relevance of the identified microbial signatures further, we performed GO and KEGG enrichment analyses based on the differentially expressed genes (DEGs) between the high- and low-risk MAPS groups. The results revealed that these DEGs were predominantly enriched in immune-related biological processes, such as immune response, cytokine activity, and chemokine signaling, indicating that the microbiome may influence host immunity and tumor behavior through immune regulation. In addition, the KEGG pathway enrichment demonstrated strong associations with key inflammatory and tumor-related pathways, including the TNF signaling, NF-kappa B, and IL-17 pathways. These findings suggest that the prognostic value of MAPS is not merely correlative but may reflect real biological mechanisms through which the microbiota contribute to tumor immune evasion or immune activation. This biologically grounded evidence further supports the hypothesis that circulating microbiomes may serve as a bridge connecting systemic immune dysregulation and tumor progression in cervical cancer.

In this study, some of the microbial genera constituting the MAPS have been previously reported in various studies. Emerging evidence suggests that certain intracellular bacteria may influence tumorigenesis by modulating the host immune system and altering the tumor microenvironment [17]. The observed variation in the abundance of *Catonella* is consistent with findings from oral cancer microbiome research, reinforcing the potential association between the oral microbiota and tumor development [32,33]. As a typical oral commensal, *Catonella* may gain access to distant anatomical sites, such as the cervix, through hematogenous dissemination, particularly under conditions of mucosal injury (e.g., oral ulcers or periodontitis), thereby contributing to local immune dysregulation and a pro-tumorigenic niche. Similarly, *Mastadenovirus* has been extensively documented in association with respiratory tract infections (particularly in pediatric populations), conjunctivitis, gastroenteritis, and urinary tract infections, and it possesses the capacity to cause severe systemic infections in immunocompromised individuals [34]. Notably, its detection in fecal samples suggests possible colonization in the gut, supporting the hypothesis that it may influence the cervical microenvironment via the gut–reproductive tract axis [35,36]. This axis, which is gaining increasing attention in microbiome research, describes the immunological and microbial interplay between the gut and reproductive systems [37], raising the possibility that *Mastadenovirus* may contribute to cervical cancer progression by modulating local or systemic immunity. The remaining microbial genera identified in this study have not yet been reported in disease-related contexts. Nevertheless, their presence in cervical cancer patients warrants further investigation to elucidate their potential roles in tumor biology and prognosis.

Despite the promising performance of the MAPS in predicting cervical cancer prognosis, certain limitations remain. Firstly, the lack of independent datasets hinders the validation of our findings. Secondly, the potential contamination inferred from the sequencing data may compromise the reliability of the dataset. Furthermore, variations in the microbiome analysis techniques could introduce bias, and the possibility of false positives cannot be ruled out. These factors may affect the robustness and generalizability of the results. To address these potential biases, it is essential to adopt standardized protocols for microbiome analysis and validate the findings using independent, multicenter datasets. Therefore, there is an urgent need for comprehensive, prospective, multicenter studies in the future to validate the prognostic utility of circulating microbiome features further and mitigate these potential biases.

In summary, this study preliminarily reveals the correlation between circulating microbiome features and the tumor microenvironment, as well as prognosis, in patients with cervical cancer. As an increasing number of microorganisms have been confirmed to be closely associated with the development and progression of various malignancies [38,39,40,41,42,43], the microbiome demonstrates significant potential in cancer research, positioning it as a key focus for future studies. The contribution of the microbiome to cancer biology is expected to become a pivotal area of cancer research in the next decade [44].

## 4. Materials and Methods

### 4.1. Data Collection

We acquired data from all patients with cervical cancer (*n* = 240) with available circulating microbial data, tumor transcriptomic data, and survival information across all stages and grades. The transcriptomic data were obtained from The Cancer Genome Atlas (TCGA) database (https://portal.gdc.cancer.gov/projects/TCGA-CESC (accessed on 1 July 2024)).

The microbiome data were derived from prior cancer microbiome studies, including whole-genome and transcriptomic sequencing data for cervical cancer from TCGA [45]. Advanced data processing tools were employed to minimize the sample contamination, ensuring that the data were solely based on whole-transcriptome sequencing results [46]. The final dataset comprised paired circulating microbiome and transcriptomic data, along with survival and clinical information from the samples.

### 4.2. The Construction of the Circulating Microbial Abundance Prognostic Score (MAPS) Model

Using the cervical cancer blood microbiome data from TCGA on 240 samples, circulating microbial prognostic features were selected, and a robust prognostic model was constructed using the “survival” (version 3.7.0), “glmnet” (version 4.1.8), and “survminer” (version 0.4.9) packages in R (version 4.4.0). Candidate microbial features significantly associated with overall survival (OS) were identified through a univariate Cox regression analysis (*p* < 0.05), followed by Lasso regression with 10-fold cross-validation to refine the feature set. Multivariate Cox regression was then applied to selecting independent prognostic features (*p* < 0.05), and the circulating Microbial Abundance Prognostic Score (MAPS) was calculated for each patient by linearly combining the OS-related microbial abundance with the multivariate Cox regression coefficients:

$$MAPS=\sum_{i=1}^{15}\left(risk\;coefficient\;of\;microbe\;i\right)\times \left(abundance\;of\;microbe\;i\right)$$

For details on the Cox regression and Lasso models, see Supplementary Materials S1.

The optimal cutoff value for stratifying the patients into the high-MAPS and low-MAPS groups was determined using the “maxstat” (version 4.1.8) package in R. The predictive performance of the MAPS for overall survival (OS) was evaluated using Kaplan–Meier curves and a receiver operating characteristic (ROC) analysis. Subsequently, a clinical correlation analysis was performed by integrating the collected clinical features (age, M stage, N stage, tumor stage, and status) with the MAPSs.

To facilitate the prediction of the 1-year, 3-year, and 5-year overall survival (OS) rates in patients with cervical cancer, a prognostic nomogram model was developed by integrating the clinical features (age, M stage, and tumor stage) with the MAPSs using the “rms” (version 6.8.1) package in R. The performance of the model was assessed using a time-dependent receiver operating characteristic (ROC) analysis and the concordance index (C-index).To characterize the landscape of the tumor microenvironment in the patients in the high-MAPS and low-MAPS groups, matched tumor transcriptomic data from these two subtypes were analyzed. The “DESeq2” (version 1.44.0) package was used to identify totally differentially expressed genes (DEGs) between the groups. Subsequently, immune-related DEGs were filtered based on the ImmPort database (https://www.immport.org/home (accessed on 15 July 2024)). Furthermore, an enrichment analysis was conducted to functionally annotate the immune-related differentially expressed genes (DEGs) and explore their involvement in biological processes and pathways. The analysis was performed using the Database for Annotation, Visualization, and Integrated Discovery (DAVID) platform (https://david.ncifcrf.gov/tools.jsp (accessed on 15 September 2024)), which provides comprehensive functional interpretations of large gene lists. This approach helps to elucidate the biological significance of immune-related DEGs and their potential contributions to cervical cancer’s progression and immune regulation.

### 4.3. Prognostic and Correlation Analyes of the Immune-Related DEGs

To comprehensively assess the prognostic significance of immune-related differentially expressed genes (DEGs) in cervical cancer, we conducted both a univariate Cox regression analysis and a Kaplan–Meier survival analysis. Univariate Cox regression was performed to identify DEGs significantly associated with overall survival, and Kaplan–Meier survival curves were generated to visualize the differences in survival between groups with high and low expression of key DEGs. The log-rank test was used to determine the statistical significance. Furthermore, we explored the potential associations between immune-related DEGs and circulating microbial prognostic features. A Spearman’s correlation analysis was employed to evaluate the relationships between the DEG expression levels and microbial abundance, aiming to uncover potential interactions between the immune landscape and the microbiome in cervical cancer prognosis. These findings provide insights into the interplay between host immune responses and microbial signatures, which may contribute to personalized therapeutic strategies.

### 4.4. The Immune Infiltration Analysis

CIBERSORT, which provides expression data for 22 immune cell types and functional states (LM22), was used to convert the gene expression data from TCGA’s cervical cancer samples into the immune cell composition. CIBERSORT allows for precise estimations of the relative abundance of different immune cell types within tumor samples, helping to reveal the characteristics of the immune microenvironment. In this analysis, we stratified the cervical cancer samples into high- and low-MAPS groups and performed an immune infiltration analysis.

By comparing the immune infiltration characteristics between the high- and low-MAPS groups, we were able to explore the potential role of the immune cells in cervical cancer and evaluate the impact of the abundance of different immune cell types on patient prognosis. A further analysis revealed differences in the distribution of the immune cells in the tumor microenvironment, which may be closely associated with tumor immune evasion mechanisms and therapeutic responses.

### 4.5. The Drug Sensitivity Analysis

In this study, the predictive value of the MAPS for the efficacy of immunotherapy and chemotherapy was explored. Tumor Immune Dysfunction and Exclusion (TIDE) scores were used to predict the immunotherapy responses for each patient. The “oncoPredict” (version 1.2) package in R was employed to estimate the half-maximal inhibitory concentration (IC50) of the drugs and predict the drug sensitivity for each patient.

### 4.6. The Statistical Analysis

The statistical analyses and graphical visualizations were performed using R (version 4.4.0) and Sangerbox (http://sangerbox.com/home.html (accessed on 15 October 2024)). Comparisons of continuous variables between groups were conducted using the *t*-test or Wilcoxon’s rank-sum test. A *p*-value < 0.05 was considered statistically significant (two-tailed).

## 5. Conclusions

This study successfully identified 15 circulating microbial genera significantly associated with patient survival and constructed a circulating Microbial Abundance Prognostic Score (MAPS) model which demonstrated a robust performance in predicting the prognosis of patients with CESC. Additionally, the integrated analysis preliminarily revealed potential interaction mechanisms between features of the circulating microbiome and the tumor immune microenvironment. A functional enrichment analysis of DEGs between the MAPS-defined groups revealed immune-related biological pathways, suggesting potential immunomodulatory roles of the microbiota. Furthermore, the integration of the MAPS into a nomogram analysis alongside the M stage and tumor stage improved the predictive accuracy of the model, further supporting the clinical utility and independent prognostic value of the MAPS. The drug sensitivity analysis further indicated that the MAPS model holds significant potential as a biomarker for predicting the efficacy of chemotherapy in patients with CESC. These findings provide novel insights into the interactions among microbiota, tumors, and the immune system, offering valuable theoretical references for precision medicine research and individualized treatment strategies for CESC.

## Figures and Tables

**Table 1:** Multivariate Cox regression results for the 15 circulating microbial genera in the MAPS model.

Microorganisms	*p*-Value	Coefficient	HR (95%CI)
*Mastadenovirus*	<0.001	1.073946	2.93 (1.6420–5.2174)
*Lambdapapillomavirus*	0.0011	1.002244	2.72 (1.4933–4.9704)
*Stanieria*	0.0069	0.820008	2.27 (1.2531–4.1141)
*Staphylothermus*	0.003	−1.05276	0.35 (0.1742–0.6990)
*Oceanimonas*	0.0055	−0.45548	0.63 (0.4596–0.8749)
*Anaplasma*	0.0245	−0.8252	0.44 (0.2135–0.8991)
*Aureimonas*	<0.001	−0.7516	0.47 (0.3182–0.6990)
*Catonella*	0.0071	0.445796	1.56 (1.1289–2.1605)
*Xylella*	0.0048	−1.13326	0.32 (0.1464–0.7080)
*Amycolatopsis*	0.0142	0.296814	1.35 (1.0613–1.7059)
*Rhodothermus*	0.0425	0.503593	1.65 (1.0171–2.6919)
*Apibacter*	<0.001	−0.89583	0.41 (0.2471–0.6746)
*Saccharomonospora*	0.0443	−0.50069	0.61 (0.3721–0.9873)
*Halonatronum*	<0.001	1.410899	4.1 (2.0107–8.3588)
*Leifsonia*	0.0148	0.582502	1.79 (1.1208–2.8603)

**Table 2:** Immune-related differentially expressed genes in cervical cancer.

Genes	*p*-Value	HR (95%CI)
*CHIT1*	0.01631	0.71 (0.5346–0.9385)
*CXCL2*	<0.001	1.02 (1.009–1.021)
*CXCL8*	<0.001	1.01 (1.003–1.007)
*EPGN*	0.002013	1.07 (1.023–1.108)
*EREG*	0.0001732	1.12 (1.055–1.187)
*FAM3B*	0.02064	0.96 (0.9297–0.994)
*IL1A*	<0.001	1.02 (1.013–1.036)
*IL1B*	<0.001	1.04 (1.02–1.051)
*IL12A*	0.004497	0.46 (0.2687–0.7857)
*STC1*	0.004388	1.01 (1.003–1.015)
*TNF*	<0.001	1.09 (1.048–1.123)

## Data Availability

The datasets are available from TCGA. The following are links to the data used in this research: https://portal.gdc.cancer.gov/projects/TCGA-CESC (accessed on 1 July 2024) and https://github.com/knightlab-analyses/mycobiome (accessed on 5 July 2024).

## Supplementary Materials

The following supporting information can be downloaded at: https://www.mdpi.com/article/10.3390/ijms26094293/s1.
