# Supplementary material for: The Significance of Circulating Microbial Signatures in the Prognosis and Immune Microenvironment of Patients with Cervical Cancer
Source: Int J Mol Sci. 2025 May 1;26(9):4293. doi: 10.3390/ijms26094293 (PMC12072589; doi:10.3390/ijms26094293)
Supplement: Supplementary file 1 [file ijms-26-04293-s001.zip › ijms-3537356-supplementary.pdf]

Supplementary Materials

The Significance of Circulating Microbial Signatures in the Prognosis and Immune Microenvironment of Patients with Cervical Cancer

Huakai Wen [1], Yumeng Zhang [1], Yongwei Liu [1], Haixia Long [2] and Yuhua Yao [1,3,4,*]

[1] School of Mathematics and Statistics, Hainan Normal University, Haikou 570100, China
[2] College of Information Science Technology, Hainan Normal University, Haikou 571158, China
[3] Key Laboratory of Data Science and Intelligence Education, Ministry of Education, Hainan Normal University, Haikou 570100, China
[4] Key Laboratory of Computational Science and Application of Hainan Province, Hainan Normal University, Haikou 570100, China
* Correspondence: yaoyuhua@hainnu.edu.cn

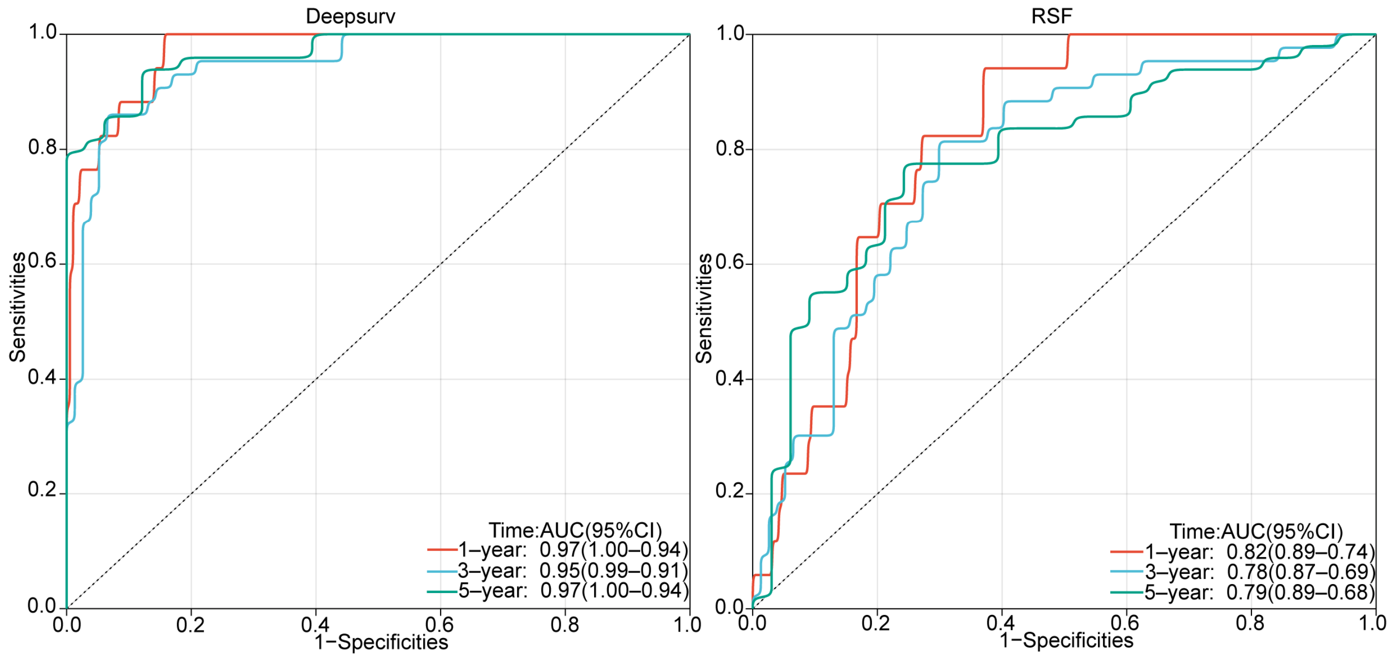

Figure S1. Time-dependent ROC Curves for Deepsurv and RSF. The AUC of the Deepsurv model for 1-year, 3-year, and 5-year were 0.97, 0.95, and 0.97; The AUC of the RSF model for 1-year, 3-year, and 5-year were 0.82, 0.78, and 0.79.

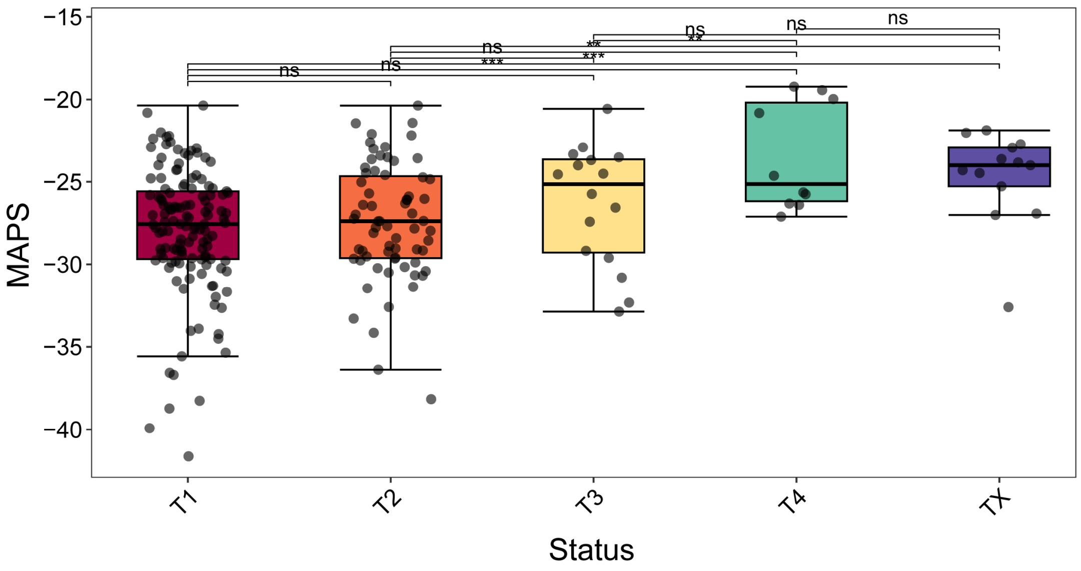

Figure S2. Differences in clinical features between the two groups were analyzed. The result revealed that MAPS groups were significantly correlated with pathological T stage (*** $p < 0.001$, ** $p < 0.01$, ns = not significant, $p > 0.05$).

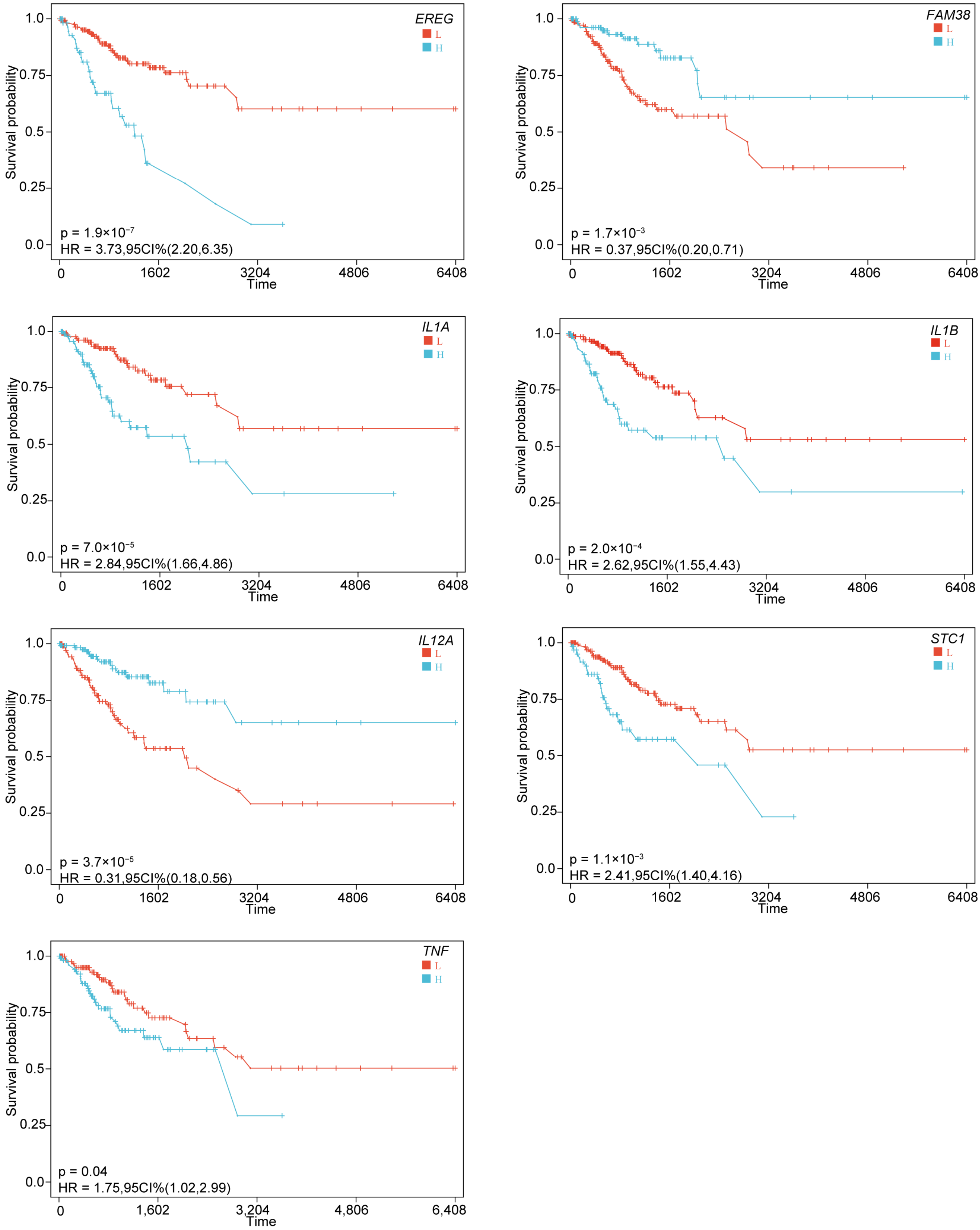

Figure S3. Survival Curve of Immune-Related Genes. The figure presents survival curves for *EREG, FAM3B, IL1A, IL1B, IL12A, STC1* and *TNF*.

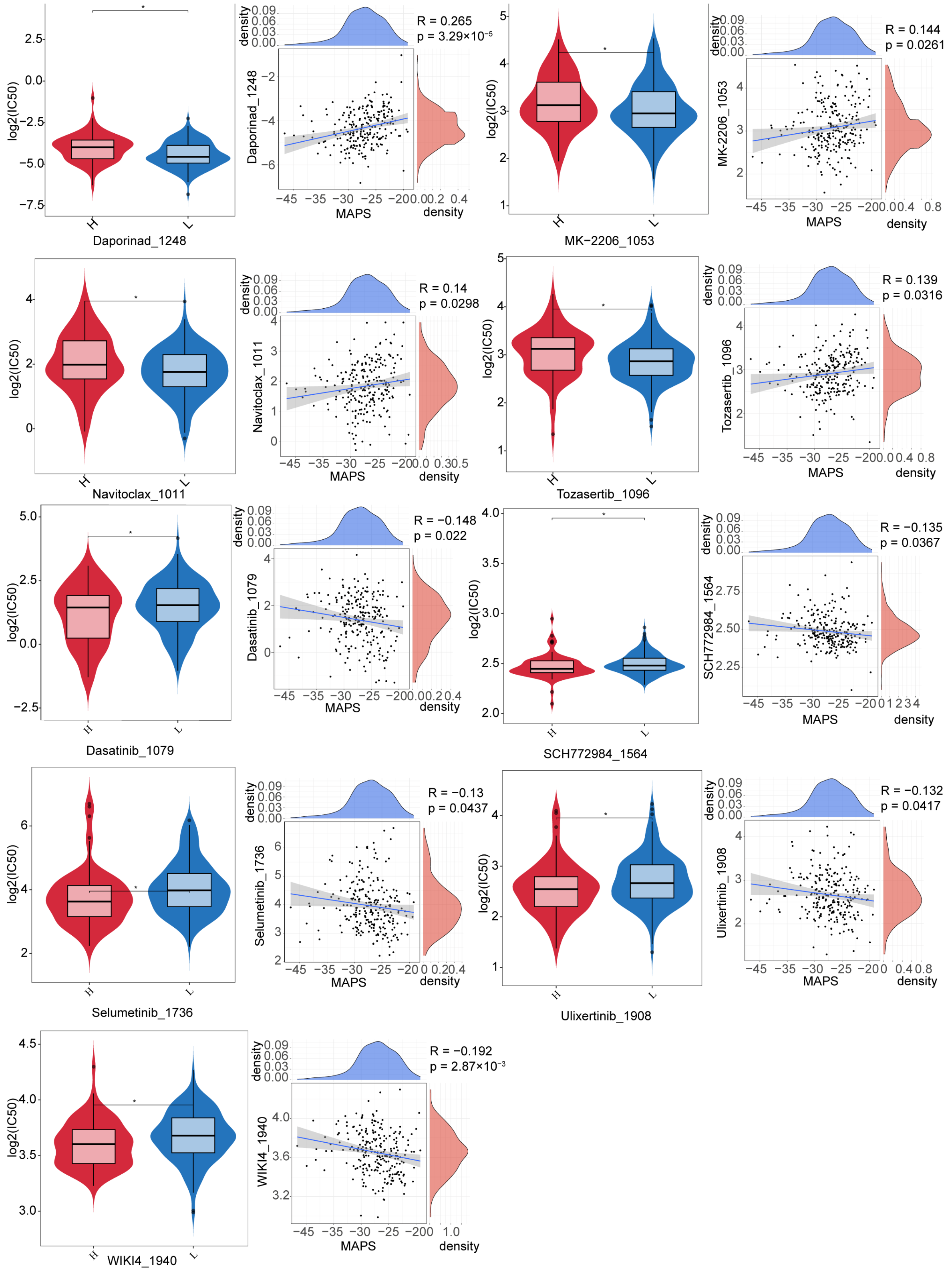

Figure S4. Box plots and correlation plots comparing the IC50 values of drugs between the MAPS high and low groups (* $p < 0.05$). The figure shows significant differences in the IC50 values of drugs *Daporinad_1248*, *Dasatinib_1079*, *MK-2206_1053*, *Navitoclax_1011*, *SCH772984_1564*, *Selumetinib_1736*, *Tozasertib_1096*, *Ulixertinib_1908* and *WIKI4_1940* between the MAPS high and low groups.

Supplementary Material S1

Overview of Cox Regression and LASSO for Prognostic Modeling

To identify prognostic microbial features associated with patient survival, we utilized the Cox proportional hazards regression model, a widely used method in survival analysis. This model estimates the hazard ratio for each variable, reflecting its effect on the risk of an event (e.g., death) over time, without requiring assumptions about the baseline hazard function.

Cox model is expressed as follow:

$$h(t \mid X) = h_0(t)\exp(\beta_1 X_1 + \beta_2 X_2 + \cdots + \beta_p X_p)$$

where $h(t \mid X)$ represents the hazard function at time t given covariates X , $h_0(t)$ is the baseline hazard function, and $\beta_1, \beta_2, \cdots \beta_p$ are the regression coefficients for the respective covariates.

However, when dealing with high-dimensional data such as microbial abundance, traditional Cox regression may suffer from overfitting, and the model's interpretability can be compromised. To address this issue, we introduced the Least Absolute Shrinkage and Selection Operator (LASSO) regression technique. LASSO is a regularization method that imposes an absolute value penalty on the regression coefficients, effectively reducing model complexity while performing feature selection by shrinking some coefficients to zero.

The objective function for LASSO Cox regression is as follows:

$$\min_\beta \left\{ -\log L(\beta) + \lambda \sum_{j=1}^{p} |\beta_j| \right\}$$

where $\log L(\beta)$ is the log-likelihood function of the Cox model, β is the vector of coefficients, λ is the regularization parameter that controls the penalty intensity. is the regularization parameter that controls the penalty intensity.

In this study, we first identified candidate microbial features significantly associated with overall survival (OS) through univariate Cox regression analysis (P < 0.05). Subsequently, we refined the feature set using LASSO Cox regression with 10-fold cross-validation and then applied multivariate Cox regression to select independent prognostic features. Based on this, the Circulating Microbial Abundance Prognostic Score (MAPS) model was constructed.

Supplementary Material S2

Overview of Random Survival Forest for Prognostic Modeling

We employed the Random Survival Forest (RSF) algorithm, a robust non-parametric ensemble learning method suitable for right-censored survival data. RSF is an extension of the traditional random forest algorithm adapted for survival analysis. It builds multiple decision trees using bootstrap sampling, and at each node, the optimal split is determined based on survival differences to enhance model prediction accuracy.

In an RSF model, each tree is grown on a bootstrap sample of the original dataset, and at each node, a random subset of variables is selected to identify the best split, typically based on maximizing survival differences (e.g., log-rank statistics). For a given sample iii, its cumulative hazard function (CHF) is estimated by averaging the CHF estimates from all out-of-bag (OOB) trees that include this sample:

$$\hat{H}(t|\ x_i) = \frac{1}{B}\sum_{b=1}^{B}\hat{H}_b(t|\ x_i)$$

Where $\hat{H}_b(t|\ x_i)$ denotes the CHF estimated by the b-th tree for sample x_i and B is the total number of trees in the forest.

In this study, we used 15 microbial features identified from multivariate Cox regression as input variables to construct the RSF model. We set the number of decision trees to 1000 and evaluated model performance using out-of-bag (OOB) error estimates. In addition, time-dependent ROC curves were employed to assess the prognostic predictive value of the model.

Supplementary Material S3

Overview of Deepsurv for Prognostic Modeling

DeepSurv leverages the representational power of neural networks to model complex, non-linear interactions between covariates and survival risk, making it well-suited for biomedical data such as microbial abundance.

The DeepSurv model estimates the hazard function as:

$$h(t|\ x) = h_0(t)\exp\left(f_\theta(x)\right)$$

where $f_\theta(x)$ is a neural network with parameters θ that outputs a risk score for input feature vector x, replacing the linear combination of covariates used in the traditional Cox model.

The network is trained by minimizing the negative partial log-likelihood function of the Cox model:

$$L(\theta) = -\sum_{i:\delta_i=1}\left(f_\theta(x_i) - \log\sum_{j\in R_i}\exp(f_\theta(x_j)) \right)$$

Where δ_i indicates whether the event was observed for subject i and R_i denotes the risk set at time t_i

In this study, we input the 15 microbial features identified from multivariate Cox regression into the DeepSurv model. The neural network was trained using the Adam optimizer with early stopping based on validation loss to avoid overfitting. After training, the model generated individualized **riskscores**, which were subsequently used for survival prediction and group stratification. The prognostic performance of the DeepSurv-derived risk score was evaluated using the time-dependent ROC curves.
